# Exploring the Evolution of Virulence Factors through Bioinformatic Data Mining

**DOI:** 10.1128/mSystems.00162-19

**Published:** 2019-05-21

**Authors:** Andrew C. Doxey, Michael J. Mansfield, Briallen Lobb

**Affiliations:** a Department of Biology, University of Waterloo, Waterloo, Ontario, Canada

**Keywords:** bioinformatics, microbial genomics, molecular evolution, pathogens, virulence factors

## Abstract

The molecular evolution of virulence factors is a central theme in our understanding of bacterial pathogenesis and host-microbe interactions. Using bioinformatics and genome data mining, recent studies have shed light on the evolution of important virulence factor families and the mechanisms by which they have adapted and diversified in function. This perspective highlights three complementary approaches useful for studying the molecular evolution of virulence factors: identification and analysis of virulence factor homologs, detection of adaptations or functional shifts, and computational prediction of novel virulence factor families. Each of these research directions is associated with distinct questions, approaches, and challenges for future work. Moving forward, bioinformatics will continue to play a critical role in exploring the evolution of virulence factors, including those that target humans. By reconstructing past processes and events, we will be able to better interpret newly sequenced microbial genomes and detect future pathoadaptations.

## Abstract (precis)

The molecular evolution of virulence factors is a central theme in our understanding of bacterial pathogenesis and host-microbe interactions. Using bioinformatics and genome data mining, recent studies have shed light on the evolution of important virulence factor families and the mechanisms by which they have adapted and diversified in function.

## PERSPECTIVE

Pathogenic microorganisms interact with hosts by producing specialized virulence factor (VF) proteins that are capable of interacting with or disrupting host processes. Due to the immense biomedical relevance of virulence factors in human infectious disease, there is a long history of research into their biology, which has revealed a remarkable diversity and sophistication in terms of structure, specificity, and mode of action. Many virulence factors are evolutionarily fine tuned to interact with and disrupt specific receptors, pathways, cell types, tissue types, and host species. This raises important evolutionary questions such as the following. How do virulence factors originate, diversify, and adapt over time? How can we use this knowledge of virulence factor evolution to discover novel virulence factors within the vast and growing collection of sequenced microbial genomes?

With the growing availability of genomes across the tree of life, it has become increasingly possible to perform comprehensive and detailed analyses of virulence factor diversity and evolution. A critical first step in many such approaches is the construction and curation of VF databases (1). The virulence factor database (VFDB), which is derived largely from a set of 74 bacterial pathogen genomes, currently contains 1,074 virulence factors. However, the VFDB also contains an additional 32,312 related VFs that can be computationally detected in genomes through homology (1). When factoring in additional pathogenic species, as well as the growing repertoire of secreted effectors, peptides, and other virulence-related molecules found in different host-pathogen systems (2), this number likely grows by orders of magnitude. Given such a vast quantity of data and possibilities for analysis, a useful schema is to divide bioinformatic analysis of VF evolution into the three conceptual approaches discussed below.

### Identifying VF homologs: exploring the sequence space near characterized VFs.

New virulence factors are typically predicted based on detectable homology to previously characterized VF sequences. Newly detected VF homologs can be of great value, as they can expand the known “sequence space” of a VF family, identify VFs in unexpected taxa, and even identify distant relationships between seemingly unrelated VF families. Detected homologs may vary considerably in their sequence similarity to a reference VF, from near-identical orthologs to remote homologs with low sequence similarity. Therefore, it can be challenging to draw the line between where one family ends and another begins, as well as to understand how sequence divergence relates to conservation of VF function.

**FIG 1:**
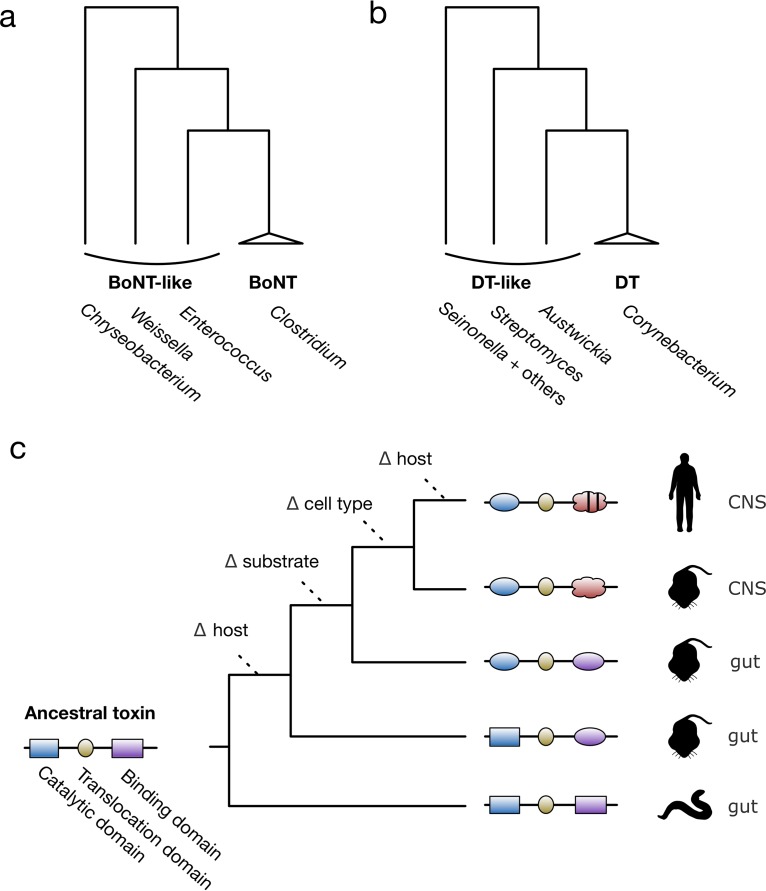
Bioinformatically identified toxins provide insights into the evolutionary origins of major toxin families. (a and b) General overviews of the phylogenies of botulinum neurotoxin (BoNT) and diphtheria toxin (DT), including recently discovered homologs. (c) General model depicting the hypothetical evolution of an ancestral toxin that diversifies over time in terms of host, cell type, and substrate specificity. Changes in the catalytic domain (blue) are associated with alterations in substrate specificity, while changes in the binding domain (purple or red) are associated with alterations in host specificity and cell/tissue type (e.g., from the gut to the central nervous system [CNS]). This model is one potential explanation for observed sequence patterns in these toxin families.

Homology-based prediction of VFs in genomes has underpinned a number of recent studies by our lab exploring the molecular evolution and diversity of bacterial exotoxins, a major class of VFs. Well-known examples include the botulinum neurotoxins (BoNT) and tetanus neurotoxins (TeNT) produced by various *Clostridia* as well as the diphtheria toxin (DT) produced by members of the *Corynebacterium* genus. BoNT and DT are also notable as the first bacterial toxins to be discovered, dating back to the late 1800s (3). Primarily through applications of PSI-BLAST, we recently identified the first homologs of these toxin families outside their respective bacterial lineages (4–6). The botulinum neurotoxin-like toxins occur outside the *Clostridium* genus in bacteria such as *Weissella* and *Enterococcus* and phylogenetically cluster nearby but outside the characterized BoNT tree (Fig. 1a). Similarly, DT-like toxins occur outside *Corynebacterium*, primarily in other actinobacterial organisms such as *Austwickia* and *Streptomyces*. These DT-like toxins also phylogenetically cluster nearby but outside the DT clade (Fig. 1b).

The identification of divergent homologs of these human disease-causing toxins has intriguing evolutionary implications. Their occurrence in bacteria not known for causing human infectious disease suggests that they may target different host species “in the wild” (e.g., the genome of Austwickia chelonae encodes a DT-like toxin, and this organism is associated with infectious lesions in reptiles rather than humans) (7). Furthermore, the sequence divergence and phylogenetic position of BoNT-like and DT-like toxins suggest that they potentially represent ancient lineages that predate the emergence of the well-known forms associated with human disease.

A major drawback of identifying toxins from sequence information is that it is difficult to predict their specific functional properties, such as host and substrate specificity (3); thus, it remains unclear how host specificity maps onto the evolutionary history of these toxin families. Once established, this information may help elucidate key evolutionary adaptations through which ancestral BoNT-like and DT-like toxins acquired specificity for different hosts, including humans.

### Detecting VF adaptations and functional shifts.

Mapping the evolution of function and specificity within VF families is an important and highly challenging research objective (Fig. 1c). Here, it is important to consider that host-pathogen arms races may drive accelerated evolution of VFs, as well as diversification of different aspects of VF function, including host, cell type, and substrate specificity. State of the art methods for exploring the evolution of specificity involve an integration of experimental and computational approaches and also rely heavily on structural biology (8). However, the ability to reconstruct such events also depends largely on the evolutionary timescale associated with the sequences being analyzed.

One exquisite example of VF adaptation is the molecular evolution of typhoid toxin. Structural studies of typhoid toxin have revealed the basis for its specificity toward humans. Unique amino acid substitutions in its glycan-binding domain allow the typhoid toxin to selectively bind human glycans terminated in Neu5Ac over glycans terminated in Neu5Gc, which are produced by other mammals (9). Although similar patterns of adaptive evolution have likely occurred in the binding domains of BoNT-like and DT-like toxins throughout their history (Fig. 1), it is difficult to pinpoint the key specificity-determining substitutions in these cases because evolutionary distances are large. In cases like these, methods that detect positive selection involving multiple substitutions may be required. For example, we developed an approach to detect spatially clustered substitutions in protein structure (10). This approach was successful in predicting ancestral adaptations within protein structures, such as the gain of a cellulose-binding site in a pathogenesis-related protein that likely contributes to its antifungal specificity (11).

Even more dramatic functional adaptations are cases in which entire segments, such as protein domains, are acquired or lost from a VF family. Recombination and domain shuffling appear to be very common in the evolution of VFs (2), and in some cases, they may even underlie the origin of new virulence functions. For example, we recently discovered a family of bacterial flagellins called flagellinolysins that have acquired a central metalloprotease domain, allowing them to localize metalloprotease activity within the external flagellar filament (12). This novel function resulting from the evolution of a new domain combination may contribute to virulence in Clostridium haemolyticum and other pathogens.

### Predicting new VF families and mechanisms.

Although the above cases explore VF diversity and evolution by starting with already characterized VF families, the computational discovery of entirely new VF families that are unrelated to existing ones is arguably the most challenging approach. One intriguing way to look for these VF sequences is to explore the thousands of domains of unknown function and “ORFan” families that have been mined from genomic and metagenomic databases (13). Because standard homology search will not work for these cases, one must rely on external information that implicates an uncharacterized protein family in virulence, such as its upregulation in virulence-associated conditions, its association with virulence phenotypes (14), its occurrence in a pathogenicity island (15), or its overrepresentation within genomes of pathogenic or host-associated species (16). Given that many virulence factors exhibit similarities to host proteins, allowing them to interfere with host functions, the identification of host-like or “mimicry” proteins in bacterial genomes is another effective strategy for virulence factor discovery (17). A recent study by Levy et al. (16) applied several of these methods to explore the genomic determinants of plant-associated bacteria. By comparing genomes of plant-associated versus non-plant-associated bacteria, Levy et al. identified thousands of plant-associated gene clusters. These clusters included new protein families that perfectly correlated with pathogenicity lifestyles, and 64 “mimicry” proteins that potentially mimic domains found in plants.

### Future directions.

With ongoing sequencing, bioinformatics will continue to expand the sequence space of VFs. This will contribute to our understanding of VF evolution and identify key sequence determinants of function. As the inventory of known VFs increases, so will our ability to predict bacterial pathogenicity from genome information, which has important implications for human health and disease. Mapping the evolution of specificity within VF and toxin families will also be critical for identifying and potentially predicting future pathoadaptations toward humans and other species. An exciting direction for future work is the use of sophisticated machine-learning approaches to discover commonalities among different virulence factor families that cannot be recognized using homology and application of these approaches to discover new VFs in genomes. Finally, through large-scale recovery of metagenome-assembled genomes, there has been an explosion of new genomic and microbiome diversity (18). New gene families encoding virulence factors and toxins can be identified in these data sets, without the need for direct culturing and experimentation (3). Through metagenomic exploration of the diversity and roles of virulence factors in host-associated microbiomes and environmental microbial communities, it will become possible to glean insights into the broader ecological roles of VFs in host-microbe interactions and ecosystem function.
